# Cases Polypus Uterus

**Published:** 1855-05

**Authors:** 


Cases of Polypus of the Womb. By Walter Channing, M. D., Boston.—This is an interesting little pamphlet in which are found thirteen cases, with details of treatment. He maintains that "*polypus is not a milignant disease*," and not necessarily fatal. He maintains that the division of polypus into *concealed* and *extra uterine* is very important, as it would necessarily modify the practice. Dr. C. is a fine writer, is peculiarly racy and highly descriptive in his style. This pamphlet should be in the hands of every medical man who aims to keep himself posted on this department, and there is no one in general practice who should not.

